# Pharmacogenetics of Efficacy and Safety of HCV Treatment in HCV-HIV Coinfected Patients: Significant Associations with *IL28B* and *SOCS3* Gene Variants

**DOI:** 10.1371/journal.pone.0047725

**Published:** 2012-11-02

**Authors:** Francesc Vidal, Miguel López-Dupla, Montserrat Laguno, Sergi Veloso, Josep Mallolas, Javier Murillas, Carmen Cifuentes, Lluis Gallart, Teresa Auguet, Gloria Sampériz, Antoni Payeras, Pilar Hernandez, Mireia Arnedo, Josep Ma Gatell, Cristóbal Richart

**Affiliations:** 1 Hospital Universitari de Tarragona Joan XXIII, IISPV, Universitat Rovira i Virgili, Tarragona, Spain; 2 Hospital Clinic, IDIBAPS, Universitat de Barcelona, Barcelona, Spain; 3 Hospital Son Espases, Palma de Mallorca, Spain; 4 Hospital Son Llàtzer, Palma de Mallorca, Spain; University of Texas Health Science Center, United States of America

## Abstract

**Background and Aims:**

This was a safety and efficacy pharmacogenetic study of a previously performed randomized trial which compared the effectiveness of treatment of hepatitis C virus infection with pegylated interferon alpha (pegIFNα) 2a vs. 2b, both with ribavirin, for 48 weeks, in HCV-HIV coinfected patients.

**Methods:**

The study groups were made of 99 patients (efficacy pharmacogenetic substudy) and of 114 patients (safety pharmacogenetic substudy). Polymorphisms in the following candidate genes *IL28B*, *IL6, IL10, TNFα, IFNγ, CCL5, MxA, OAS1, SOCS3, CTLA4* and *ITPA* were assessed. Genotyping was carried out using Sequenom iPLEX-Gold, a single-base extension polymerase chain reaction. Efficacy end-points assessed were: rapid, early and sustained virological response (RVR, EVR and SVR, respectively). Safety end-points assessed were: anemia, neutropenia, thrombocytopenia, flu-like syndrome, gastrointestinal disturbances and depression. Chi square test, Student's T test, Mann-Whitney U test and logistic regression were used for statistic analyses.

**Results:**

As efficacy is concerned, *IL28B* and *CTLA4* gene polymorphisms were associated with RVR (p<0.05 for both comparisons). Nevertheless, only polymorphism in the *IL28B* gene was associated with SVR (p = 0.004). In the multivariate analysis, the only gene independently associated with SVR was *IL28B* (OR 2.61, 95%CI 1.2–5.6, p = 0.01). With respect to safety, there were no significant associations between flu-like syndrome or depression and the genetic variants studied. Gastrointestinal disturbances were associated with *ITPA* gene polymorphism (p = 0.04). Anemia was associated with *OAS1* and *CTLA4* gene polymorphisms (p = 0.049 and p = 0.045, respectively), neutropenia and thromobocytopenia were associated with *SOCS3* gene polymorphism (p = 0.02 and p = 0.002, respectively). In the multivariate analysis, the associations of the *SOCS3* gene polymorphism with neutropenia (OR 0.26, 95%CI 0.09–0.75, p = 0.01) and thrombocytopenia (OR 0.07, 95%CI 0.008–0.57, p = 0.01) remained significant.

**Conclusions:**

In HCV-HIV coinfected patients treated with PegIFNα and ribavirin, SVR is associated with *IL28B rs8099917* polymorphism. HCV treatment-induced neutropenia and thrombocytopenia are associated with *SOCS3 rs4969170* polymorphism.

## Introduction

Patients who are co-infected with hepatitis C virus (HCV) within human immunodeficiency virus (HIV) are currently treated on a 48-week regimen of pegylated interferon alpha (pegIFNα) and ribavirin [1]. Although new antiviral agents that are active against HCV are now available [2], they are just becoming to be used for treating HCV-HIV co-infected patients. Response to the treatment regimen varies greatly between individuals, as it does between HCV-mono-infected individuals. Studies elsewhere have identified several factors that can independently predict treatment response: age, duration of the infection, HCV genotype, baseline plasma HCV viral load and the degree of liver fibrosis, among others [3]–[5]. These factors, however, do not fully explain the variability in the response to treatment, hence other factors, such as host genetic background, have been sought [6], [7].

Pharmacogenetics is the science that studies interindividual variations in the response to and toxicity of drugs due to variations in the genetic composition of individuals, in other words, how a person's genetic background influences the favourable or adverse outcome of a certain treatment. Sufficient advances have been made in this discipline to allow this fertile field of research to move out of the research laboratory into the scenario of potential clinical applications, including the treatment of HCV. In 2009, various independent research teams provided evidence that the *interleukin 28B (IL28B) rs12979860* and *rs8099917* polymorphisms were associated with spontaneous clearance of HCV [8] and with sustained virological response to treatment with pegIFNα and ribavirin [9]–[11] in HCV mono-infected patients. Further studies have consistently confirmed these associations [12], [13]. Similar findings have also been reported in HCV-HIV co-infected patients [14]–[17].

Recently, we carried out a randomised trial to compare the efficacy and safety of the two available forms of pegIFNα plus ribavirin in HCV-HIV co-infected patients [18]. No significant differences in either efficacy or safety were found between the two treatment arms. The present report is a pharmacogenetic substudy of that study. Here we assess the possible relationship between the efficacy and safety of pegIFNα plus ribavirin and polymorphisms in the genes that encode for several proteins involved in the metabolism of interferon α and ribavirin and in the defence against viral infections.

## Methods

### Study design and patients

This was a pharmacogenetic substudy of the PegIFNα 2a vs. PegIFNα 2b, both plus ribavirin, study (Clinical Trial Registry Number: ISRCTN81765620. Registration Number in AEMPS: 03-0198), which was a prospective, multicentred, randomised, open-label trial. Details of the study design and characteristics have been reported elsewhere [18]. From the 182 patients included in that trial, 123 had stored DNA available and constitute the basis of the current pharmacogenetic study. Of these patients, 10 had discontinued the study (2 voluntary and 8 protocol violation) and 14 had not completed the scheduled 48-week treatment regimen because of discontinuation due to severe adverse effects. Hence, 99 patients completed the study protocol (or stopped it according to standart early virological stopping rules) and had DNA available. The pharmacogenetic substudy of efficacy was performed in these 99 individuals. For the pharmacogenetic substudy of safety we assessed these 99 patients plus the 14 who had discontinued treatment because of toxicity (n = 113). Patients were evaluated before beginning treatment, 2 weeks after initiation and every 4 weeks thereafter until cessation of therapy. One last evaluation of the sustained viral response (SVR) was made 24 weeks after cessation of therapy. A complete cell count and routine biochemical tests including lactate were conducted at every medical visit, together with a medical interview in order to monitor possible secondary effects associated with treatment.

### Ethics

Participants provided written informed consent before taking part in the study. The institutional ethics committees of the participating centres specifically approved this study. The full names of the institutional review boards and committees are: Comitè Ètic d'Investigació Clínica de l'Hospital Clinic (for the Hospital Clinic, Barcelona, Spain), Comitè Ètic d'Investigació Clínica de les Illes Balears (for the Hospital Son Llàtzer and the Hospital Son Espases, from Palma de Mallorca, Spain) and Comitè Ètic d'Investigació Clinica de l'Hospital Universitari de Tarragona Joan XXIII (for the Hospital Joan XXIII, Tarragona Spain). The study protocol was in accordance with the Declaration of Helsinki of good clinical practice guidelines.

### Assessment of efficacy

The primary measure of efficacy was SVR, which was defined as undetectable plasma HCV-RNA at the end of the 24 week-period of follow-up after cessation of the scheduled 48-week treatment. Patients with detectable HCV-RNA after 24 weeks of therapy were considered failures, and therapy was discontinued. Secondary parameters of efficacy were: 1) early virological response (EVR), defined as negative HCV-RNA or a ≥2 log reduction of HCV-RNA from baseline at week 12 of treatment; 2) rapid virological response (RVR), defined as negative HCV-RNA at week 4 of treatment and, 3) relapses, defined as patients with EVR but not SVR.

### Assessment of safety

Adverse events were graded as mild, moderate, severe, or potentially life-threatening according to a modification of the World Health Organisation scale [19]. Therapy was permanently discontinued in the face of life-threatening events. In cases of haematological toxicity, the ribavirin or PegIFNα dose was lowered according to the drug label recommendations and full doses were restarted when the haematological data returned to the normal level for that patient. The use of granulocyte-colony stimulating factor and erythropoietin were permitted in this study and used at the discretion of the physician responsible for each patient, as was the use of antidepressant drugs. Anemia was defined as haemoglobin level of <10.5 g/dL. Neutropenia was defined as a neutrophile count of less than 2.5×10^9^ cells/L. Thrombocytopenia was considered when the platelet count fell below 125×10^9^ platelets/L. Patients suspected of suffering depression were evaluated by a psychiatrist, and the presence/absence of depression was assessed by the Structured Clinical Interview for DSM-IV axis 1 Disorders (SCID) [20]. Adverse gastrointestinal effects were considered if nausea, vomiting and/or abdominal pain were present. Flu-like symptoms considered were fatigue, fever, myalgia and headache.

### Laboratory methods

#### Samples

After an overnight fast, 20 mL of blood was collected from an antecubital vein into a Vacutainer™ with ethylene diamine tetra-acetic acid (EDTA). Five mL of whole blood was used to determine CD4+ T-cell count. Five-hundred µL was used for DNA isolation by a MagNa Pure LC Instrument (Roche Diagnostics, Basel, Switzerland). Plasma and serum were obtained by centrifugation at 3500 g for 15 min at 4°C.

#### Measurements

HCV infection was assessed by detection of a positive anti-HCV antibody test in serum, through indirect qualitative immunoassay (sandwich twice washed) (Advia Centaur, Bayer Health Care, Tarrytown, NY). Plasma HCV viral load was determined by a quantitative polymerase chain reaction assay (Versant HCV-NA 3.0 {bDNA}, Siemens Medical Soluctions Diagnostics, Tarrytown, NY). HCV genotyping was carried out as previously described [21]. HIV-1 infection was diagnosed by a positive enzyme-linked immunoabsorbent assay and confirmed by a positive Western blot test. Plasma HIV-1 viral load was determined by the Cobas amplicor HIV-1 Monitor Test v 1.5 using the Cobas Amplicor system (Roche Diagnostics, City, State/Country). CD4 T-cell count was assessed in a flow cytometer FAC Scan (Becton Dickinson Immunocytometry Systems, San Jose, CA). Data acquired was analysed using the Multiset program.

#### Genetic analyses

The nomenclature and details of the single nucleotide polymorphisms (SNPs) assessed are shown in Table 1. We selected to assess SNPs in genes encoding for: a) several cytokines (IL28B, IL6, IL10, TNFα and IFNγ) given that they are involved in the host immune respose to HCV; b) the chemokine CCL5, because of its expression is enhanced by HCV; c) the proteins MxA, OAS1 and SOCS3, which regulate the potent antiviral effect of interferon α; d) the cytotoxic lymphocyte antigen CTLA4, that modulate the response of HCV to interferon α, and; e) ITPA, since it has been associated with anemia in patients treated with purine analogues. Genetic analyses were carried out in the Centro Nacional de Genotipado (CeGen), Spain (www.cegen.org). The methodology applied in the genotyping was the single-base extension polymerase chain reaction Sequenom iPLEX-Gold.

**Table 1:** Genes and polymorphisms assessed.

Gene	rs	Chromosome
*IL28B*	rs8099917	19
*IL6*	rs1800795	7
*IL10*	rs1800872	1
*IL10*	rs1800896	1
*TNFα*	rs361525	6
*IFNγ*	rs2430561	12
*CCL5*	rs1800825	17
*CCL5*	rs2107538	17
*CCL5*	rs2280788	17
*CCL5*	rs2280789	17
*MxA*	rs462903	21
*OAS1*	rs2660	12
*SOCS3*	rs12952093	17
*SOCS3*	rs4969168	17
*SOCS3*	rs4969170	17
*CTLA4*	rs231775	2
*CTLA4*	rs5742909	2
*ITPA*	rs7270101	20
*ITPA*	rs1127354	20

Nomenclature (acronyms in parentheses): interleukin 28B (IL28B), interleukin 6 (IL6), interleukin 10 (IL10), tumor necrosis factor alpha (TNFα), interferon gamma (IFNγ), chemokine C ligand 5 (CCL5*), myxovirus resistance protein A (MxA), oligoadenyl synthase 1 (OAS1), suppressor of cytokine signalling 3 (SOCS3), cytotoxic T lymphocyte antigen 4 (CTLA4), inosine triphosphatase (ITPA).

* Formerly known as: regulated upon activation normally expressed and secreted (RANTES).

### Statistical analysis

A descriptive analysis of the baseline variables was conducted. Before statistical analysis, normality distribution and homogeneity of the variables were tested by the Kolmogorov-Smirnov test. Continuous variables were expressed as mean±SD or median (interquartile range), depending on its distribution, and discrete variables were expressed as percentage. Hardy-Weinberg equilibrium was assessed by the χ^2^ goodness-of-fit test. Linkage disequilibrium and haplotype analysis, after its reconstruction, were made with the Haploview program [22]. The reconstruction of haplotypes from genotype data of *IL10, CTL4, IFN-γ, OAS1, CCL5* and *ITPA* genes was performed with the PHASE v 2.1 program [23], [24]. Student's T test was used to compare normally distributed continuous variables with every type of virological response (RVR, EVR and SVR) and with every category of adverse effects. The Mann-Whitney U test was performed to compare continuous variables that were not normally distributed. Comparisons of qualitative variables, including genotype, allele frequencies, clinical, analytical and therapy variables, with the different types of virological response and with toxicity were analyzed by the Chi-square test, and Fisher's exact test when necessary. Odds ratios and confidence intervals were calculated using Woolf approximation. Logistic regression test was performed to study predictive factors of sustained viral response and toxicity, and only the variables with p<0.05 at univariate analysis were included in the multivariate analysis. The variables included in the multivariate models were selected by means of a forward-backward stepwise procedure. All analyses were performed using the SPSS/PC+ statistical package (V. 17.01 for Windows; Chicago, IL). A p value of less than 0.05 was considered significant, and all statistical tests were two tailed.

## Results

### Patients

Table 2 shows the main demographic and clinical characteristics of the 123 patients that had DNA available, of the 99 patients selected for the pharmacogenetic study of efficacy and of the 113 patients assessed for the pharmacogenetic study of safety. Demographic and clinical characteristics of these 123 subjects did not differ significantly from those of individuals who had not stored DNA available (n = 59). Of note, there was a population admixture regarding HCV genotypes (Table 2).

**Table 2:** Main demographic and clinical characteristics of the groups studied.

		All patients (n = 123)	Efficacy study (n = 99)	Safety study(n = 113)
Male gender (%)		74.8	74.7	74.3
Age (years)*		41 (38–43)	40 (38–43)	40 (38–43)
Baseline weight (Kg)**		69.3±12	69.5±12	69.3±12
Baseline height (cm)**		170.7±8.2	170.9±8.4	170.7±8.3
HCV/HIV acquiring risk factor (%)	IDU	74	72.7	72.6
	Heterosexual	17.1	18.2	17.7
	Male homosexual	5.7	6.1	6.2
	Haemophiliac	0.8	1	0.9
	Other	2.4	2	2.7
AIDS (%)		42.2	40.4	42.5
HCV genotype (%)	1	43.9	45.4	44.2
	2	3.3	3	3.5
	3	34.1	32.3	32.7
	4	18.7	19.2	19.4
Baseline HCV-RNA>400000 IU/mL (%)		78.3	77.1	77.3
Baseline HCV-RNA>600000 IU/mL (%)		60	58.3	60
Baseline HCV-RNA>800000 IU/mL (%)		57.5	55.2	57.3
Fibrosis score, (%)	0–1	34.9	35.7	35.1
	2–4	65.1	64.3	64.9
Steatosis (%)		34	28.6	34.1
Baseline CD4 cell count (cells/mL)*		551 (430–733)	562 (434–760)	562 (415–787)
Baseline CD4 cell count ≤350 cells/mL (%)		14.8	15.3	16.2
Baseline HIV viral load≤200 copies/mL (%)		78.7	75.5	77.7
HAART (%)		84.5	82.8	84.1
RVR (%)		38.4	35.4	36.3
EVR (%)		75.4	73.9	74.7
SVR (%)		49.6	51.5	50
Discontinuation for toxicity (%)		11.4	0	12.8
Toxicity (%)		92.7	90.9	92
Anemia (%)		28.5	28.3	26.5
Neutropenia (%)		39.8	40.4	40.7
Thrombocytopenia (%)		39	38.4	39.8
Flu-like syndrome (%)		69.1	69.7	68.1
Headache (%)		15.4	16.2	15.9
Depression (%)		32.5	32.3	33.6
Gastrointestinal symptoms (%)		24.4	26.3	24.8
Lactic acidosis (%)		4.1	2	3.5

* Median (25–75% interquartile range).

** Mean±standard deviation.

HAART: Highly active antiretroviral therapy. EVR: Early virological response. RVR: Rapid virological response. SVR: Sustained virological response.

### Efficacy analyses

Table 3 shows the association between the genotypes and alleles assessed and each type of virological response. RVR was associated with *IL28B rs8099917* SNP, carriers of *T* allele had the greater association with response, and with *CTLA4 rs231775* SNP, carriers of the *A* allele were associated with better response. No associations were observed with EVR. SVR was otherwise associated with the *IL28B rs8099917* SNP, carriers of the *T* allele being associated with better outcome. After analysing the linkage disequilibrium among the polymorphisms studied, only an association between *CCL5 rs2280789* and *rs2107538* SNPs was found (D′ = 1; 95%IC = 0.87–1). The reconstructed haplotypes of these polymorphisms were not associated with SVR (p = 0.14).

**Table 3:** Association between the different types of virological response and the genetic variants assessed.

Genotype/allele		RVR (n = 35)		EVR (n = 73)		SVR (n = 51)	
		OR (95%CI)	p	OR (95%CI)	p	OR (95%CI)	p
***IL28B rs8099917***	*TT*	1		1		1	
	*GT*	0.41 (0.14–1.23)	0.1	0.42 (0.15–1.21)	0.25	0.35 (0.14–0.87)	0.01
	*GG*	0		0.44 (0.04–5.39)		0	
	*T*	2.8 (1.1–7.58)	0.03	1.9 (0.84–4.46)	0.12	3.1 (1.41–6.79)	0.004
***CTLA4 rs231775***	*AA*	1		1		1	
	*AG*	0.29 (0.09–1)	0.03	0.78 (0.27–2.21)	0.46	0.36 (0.08–1.72)	0.23
	*GG*	0.13 (0.01–1.11)		3 (0.33–26.79)		0.74 (0.16–3.35)	
	*A*	3.6 (1.44–9)	0.004	0.8 (0.37–1.76)	0.6	1.16 (0.6–2.27)	0.66

RVR: Rapid virological response. EVR: Early virological response. SVR: Sustained virological response. OR: Odds ratio. CI: confidence interval.

Odds ratio in the different genotypes are referred to the genotype with OR = 1. The value “0” means that there were no subjects carrying this genotype.

Note: the remaining genetic variants assessed showed no significant associations with RVR, EVR and SVR (p>0.05 for all comparisons).

Univariate analysis of SVR, which included several clinical, virological and therapy variables, indicated that HCV genotypes 2+3, RVR and EVR, beaides of carriage of the *IL28B rs8099917 T* allele, were significantly associated with SVR (Table 4). To determine whether the *IL28B rs8099917* SNP was independently associated with SVR, we constructed a multivariate regression model which included the variables reported to be significantly associated with SVR in the univariate analysis. After adjustment for other covariates, the *IL28B rs8099917 T* allele remained significantly associated with SVR, compared with the *G* allele (Table 5).

**Table 4:** Association of clinical, biochemical, and therapeutic factors with SVR in patients who completed the scheduled 48-week treatment regimen with pegIFNα and ribavirin.

Variables	Odds ratio	95% CI	p
Male vs. female	2.33	0.91–5.96	0.07
Age ≤40 vs. >40 years	2.17	0.97–4.85	0.06
Weight<75 vs. ≥75 Kg	0.48	0.19–1.18	0.1
CDC stage B vs. A	0.66	0.2–2.17	0.49
CDC stage C vs. A	0.42	0.14–1.26	0.12
HIV viral load<200 vs. ≥200 copies/mL	1.39	0.55–3.5	0.48
HAART (yes vs. no)	0.9	0.33–2.66	0.9
ABC (yes vs. no)	1.46	0.5–4.17	0.48
HCV RNA>800000 vs. ≤800000 IU/mL	0.9	0.4–2.02	0.8
HCV RNA>600000 vs. ≤600000 IU/mL	0.69	0.3–1.56	0.37
HCV RNA>400000 vs. ≤400000 IU/mL	0.54	0.2–1.44	0.22
HCV genotype 2+3 vs. 1+4	4.65	1.85–11.6	0.001
Fibrosis 2–4 vs. 0–1	1.35	0.56–3.3	0.5
Steatosis (yes vs. no)	1.39	0.31–6.14	0.7
CD4≤350 cells/mL	2.96	0.87–10	0.07
EVR (yes vs. no)	>999.999		<0.001
RVR (yes vs. no)	31.2	6.56–148.37	<0.001
pegIFNα 2a vs pegIFNα 2b	1.4	0.65–3.17	0.37

pegIFNα: pegylated interferon alpha. CI: confidence interval. CDC: Centers for Disease Control.

HAART: highly active antiretroviral therapy. ABC: abacavir. EVR: early virological response. RVR: rapid virological response.

**Table 5:** Independent predictors of SVR in patients who completed the scheduled 48-week treatment regimen with pegIFNα and ribavirin.

Variables	Odds ratio	95% CI	p
*IL28B T* vs. *G* allele	2.61	1.2–5.6	0.01
HCV Genotype 2+3 vs. 1+4	3.61	1.98–6.58	<0.001

SVR: sustained virological response. pegIFNα: pegylated interferon alpha. CI: confidence interval.

### Safety analyses

Table 6 shows the association between the genotypes and alleles assessed and the different categories of adverse effects defined. There were no significant associations between flu-like syndrome or depression and any of the genetic variants studied. Adverse gastrointestinal disturbances were associated with the *ITPA rs1127354* polymorphism. Anemia was associated with the *OAS1 rs2*660 and the *CTL4 rs231775* polymorphisms, and neutropenia and thrombocytopenia were associated with the polymorphism *rs4969170* in the *SOCS3* gene.

**Table 6:** Association between the different types of adverse effects and the genetic variants assessed.

Genotypes and alleles		Gastrointestinal (n = 28)	p	Flu-like syndrome (n = 77)	p	Depression (n = 38)	p	Anemia (n = 30)	p	Neutropenia (n = 46)	p	Thrombocytopenia (n = 45)	p
***CTL4 rs231775***	*GG*	1		1		1		1		1		1	
	*AG*	3.6 (0.4–32.24)	0.48	2.33 (0.48–11.23)	0.31	0.35 (0.08–1.48)	0.26	0.17 (0.04–0.76)	0.045	0.59 (0.14–2.44)	0.76	0.29 (0.06–1.3)	0.12
	*AA*	2.77 (0.32–24.13)		0.79 (0.18–3.41)		0.65 (0.17–2.52)		0.25 (0.06–1.03)		0.7 (0.18–2.73)		0.23 (0.05–1.02)	
	*A*	1.18 (0.56–2.5)	0.66	0.68 (0.33–1.39)	0.29	1.06 (0.55–2.03)	0.87	0.65 (0.53–1.28)	0.21	0.93 (0.49–1.76)	0.83	0.55 (0.29–1.04)	0.07
***ITPA rs1127354***	*CC*	1		1		1		1		1		1	
	*AC*	4.14 (1.25–13.72)	0.04	2.7 (0.57–12.98)	0.15	3.79 (1.14–12.65)	0.06	0.19 (0.02–1.51)	0.18	0.83 (0.25–2.7)	0.66	0.87 (0.26–2.87)	0.48
	*AA*	0		0		0		0		0		0	
	*A*	2.76 (0.95–8)	0.054	1.28 (0.39–4.19)	0.78	2.4 (0.85–7.06)	0.09	0.17 (0.02–1.35)	0.07	0.68 (0.22–2.05)	0.49	1.23 (0.43–3.53)	0.7
***OAS1 rs2660***	*AA*	1		1		1		1		1		1	
	*AG*	0.64 (0.25–1.65)	0.18	0.56 (0.22–1.42)	0.23	1.19 (0.49–2.88)	0.88	0.35 (0.12–1)	0.049	1.29 (0.55–3)	0.81	1.4 (0.6–3.27)	0.72
	*GG*	0.18 (0.02–1.48)		0.37 (0.1–1.31)		1.33 (0.37–4.71)		1.66 (0.48–5.74)		0.94 (0.27–3.28)		1.02 (0.29–3.58)	
	*A*	2.05 (0.98–4.32)	0.06	1.77 (0.95–3.3)	0.07	0.85 (0.46–1.56)	0.59	1.06 (0.55–2.03)	0.87	0.95 (0.52–1.72)	0.86	0.89 (0.49–1.62)	0.71
***SOCS3 rs4969170***	*GG*	1		1		1		1		1		1	
	*AG*	1.1 (0.42–2.89)	0.97	1.45 (0.56–3.74)	0.09	1.51 (0.62–3.64)	0.41	0.67 (0.25–1.79)	0.51	0.28 (0.11–0.7)	0.02	0.71 (0.31–1.67)	0.005
	*AA*	0.95 (0.26–3.53)		0.4 (0.13–1.26)		0.68 (0.19–2.47)		1.34 (0.41–4.39)		0.71 (0.23–2.18)		0.06 (0.007–0.49)	
	*A*	1 (0.52–1.92)	1	0.69 (0.38–1.26)	0.23	0.95 (0.52–1.73)	0.86	1.01 (0.56–2)	0.86	0.64 (0.36–1.15)	0.13	0.38 (0.2–0.7)	0.002

Values are expressed as odds ratio (OR) and 95% confidence interval. Odds ratio in the different genotypes are referred to the genotype with OR = 1. The value “0” means that there were no subjects carrying this genotype.

Note: the remaining genetic variants assessed showed no significant associations with any of the adverse effects (p>0.05 for all comparisons).

We performed an univariate analysis of the association of the six categories of adverse effects defined and the clinical, virological and therapy variables that were assessed in the safety substudy (the variables used are detailed in Table 4). Among continuous variables, the significant associations observed were: flu-like syndrome with higher weight (71.1±12.7 vs. 65.2±9 k.; p = 0.02); depression with higher HIV plasma viral load (median 199 copies/mL, 25–75% IQR 49–298 vs. median 49 copies/mL, 25–75% IQR 39–199; p = 0.045), neutropenia with lower CD4 T-cell count (median 508 cells/mL, 25–75% IQR 340–769 vs. median 591 cells/mL, 25–75% IQR 497–800; p = 0.03) and thrombocytopenia with lower CD4 T-cell count (median 511 cells/mL, 25–75% IQR 383–704 vs. non neutropenic: median 611 cells/mL, 25–75% IQR 473–816; p = 0.049). Among categoric variables, the only significant associations observed were: neutropenia with pegIFNα 2a vs. 2b (OR 2.6, 95CI 1.2–5.7, p = 0.01), and with female vs. male gender (OR 2.7, 95%CI 1.1–6.4, p = 0.02); and thrombocytopenia with pegIFNα 2a vs. 2b (OR 2.9, 95%CI 1.3–6.3, p = 0.008).

For multivariate analyses, we constructed a logistic regression model which included the adverse effects categories that were associated with more than one clinical, analytical, virological or genetic variable in the univariate analysis. The only adverse effects that fulfilled these criteria were neutropenia and thrombocytopenia. With respect to neutropenia we constructed a multivariate regression model which included the following variables: gender, type of pegIFNα prescribed, CD4 cell count stratified in two categories (≤350 and >350 cells/mL) and *SOCS3 rs4969170* genotype. The use of pegIFNα 2a and CD4 cell count ≤350 cells/mL was associated with greater risk of neutropenia while carriers of the *SOCS3 rs4969170 AG* genotype had significantly lower risk of neutropenia. Regarding thrombocytopenia, the model included the type of pegIFNα prescribed, stratified CD4 cell count, and *SOCS3 rs4969170* genotype. Patients treated with pegIFNα 2a had significantly higher risk of thrombocytopenia, and carriers of the *SOCS3 rs4969170 AA* genotype had significantly lower risk of thrombocytopenia (Table 7).

**Table 7:** Independent predicitive factors of adverse effects.

		Neutropenia		Thrombocytopenia	
		OR (95%CI)	p	OR (95%CI)	p
PegIFNα 2a vs. 2b		4.23 (1.59–11.23)	0.04	2.62 (1.91–6.29)	0.03
Female vs. male		2.51 (0.94–6.74)	0.07	*	*
CD4 cell count ≤350 vs. >350 cells/mL		4.69 (1.29–17.24)	0.02	1.94 (0.58–6.49)	0.28
*SOCS3 rs4969170*	*GG*	1		1	
	*AG*	0.26 (0.09–0.75)	0.01	0.75 (0.31–1.83)	0.53
	*AA*	1.1 (0.28–3.9)	0.94	0.07 (0.008–0.57)	0.01

OR: odds-ratio. CI: Confidence interval. pegIFNα: pegylated interferon alpha.

* Gender was not included in multivariate analysis of thrombocytopenia, because in the univariate analysis this variable did not fulfill criteria for inclusion in the multivariate analysis.

## Discussion

Given that HCV treatment is of long duration, has potentially serious adverse effects and is expensive, several attempts have been made to identify the factors that predict a successful outcome [3]–[5]. Our previous study identified that factors associated with treatment outcome are: age, HCV viral load and HCV genotype [18]. This pharmacogenetic substudy shows that the *IL28B rs8099917* polymorphism is associated with SVR to hepatitis C therapy in HCV-HIV co-infected patients and therefore confirms the results that have been reported in many other studies performed in HCV mono-infected [9]–[13] and in HCV-HIV co-infected patients [14]–[17]. A new finding from our study is the association between HCV treatment-induced neutropenia and thrombocytopenia and the *SOCS3 rs4969170* polymorphism.

As far as cytokines are concerned and besides *IL28B*, we have assessed the effect of polymorphism in genes encoding for diverse cytokines, such as *IL6, IL10, TNFα* and *IFNγ*, given that they are involved in the immunological response to HCV [6], [7], [25], [26]. The genes that encode for these cytokines are polymorphic and genetic variants may have functional significance at the protein level. Despite this, our data do not show any significant associations between *IL6, IL10, TNFα* and *IFNγ* polymorphisms and virological response to treatment with pegIFNα and ribavirin. Our results agree, therefore, with the lack of association found between polymorphism in these cytokine-encoding genes and virological response to HCV treatment [27]–[29]. Nevertheless, the data from the present study do not confirm the positive association between virological response and *IL6* [7] and *IL10* [6], [30] polymorphisms. The reasons for this discrepancy may be due to the low number of patients assessed in some investigations [6], [7], [30] as well as in the current study, which means unstable and, often, non-replicable data. Differences in the type of population assessed (HCV mono-infected vs. HCV-HIV co-infected) and in the type of HCV treatment used (interferon monotherapy vs. pegIFNα plus ribavirin) may offer additional explanation.

With respect to chemokines, we have assessed CCL5. The expression of CCL5 is enhanced in liver and in blood by HCV and successful HCV treatment supressess this upregulation [31]. Previous studies in HCV monoinfected patients have shown that *CCL5 rs2107538* SNP and some *CCL5* haplotypes are associated with HCV treatment response [32], [33] although data are inconsistent [34]. Of note, several patients in these studies were treated with standard interferon α rather than with pegIFNα. The current study is the first one performed in HCV-HIV coinfected subjects and our data suggest no relationship between *CCL5* gene polymorphisms and SVR. Haplotyping confirmed this lack of association. Differences between our results and those provided by other investigations [32], [33] may be searched in the population assessed (HCV monoinfected vs. HCV-HIV coinfected) and/or in the type of interferon used (standart interferon α vs. pegIFNα). Furthermore, no association was observed between *CCL5* gene polymorphisms and the different types of adverse effects assessed.

We also assessed the role of polymorphisms in the interferon pathway given that IFNα increases the expression of several genes involved in the immunological response to HCV [35]. IFNα has a potent antiviral action that is exerted indirectly through a complex mechanism [36] in which the myxovirus resistance protein A (MxA), the oligoadenylate synthase 1 (OAS1) and the suppressor of cytokine signaling 3 (SOCS3) are involved [37]. The genes that encode for MxA, OAS1 and SOCS3 are polymorphic and it has been assessed whether polymorphism in these genes modulate the response to interferon in HCV monoinfected subjects [38], [39]. The *SOCS3 −487A* allele increases SOCS3 expression and was associated with pegIFNα and ribavirin HCV treatment failure [38]. Furthermore, carriage of the *MxA* −*88G>T* allele, was associated with a better response of HCV to interferon and polymorphism located in *OAS1* gene was shown to be associated with spontaneous HCV clearance [39]. Our data in the current study in HCV-HIV coinfected subjects indicates that *MxA, OAS1* and *SOCS3* SNPs are not associated with HCV treatment efficacy and therefore confirm the results of previous investigations [40]. A new finding from our study was the association of the *SOCS3 rs4969170* polymorphism with HCV treatment-induced neutropenia and thrombocytopenia. Plausible biological explanation can be searched in the fact that in studies in knockout mice, SOCS3 has been shown to be implicated in both granulopoiesis [41] and thrombopoiesis [42].

CTLA4 is a polypeptide involved in the processing of antigens by T-cell lymphocytes and influences the response of HCV to interferon. Three studies have assessed the relationship between the polymorphisms *rs231776* (+*49A>G*) and *rs5247909* (*−318C*>*A*) in the *CTLA4* gene and HCV treatment response, either in HCV monoinfected patients [43], [44] and in HCV/HIV co-infected individuals [45]. Despite some discrepancies regarding the effect of gender or the type of interferon α used, data from these studies were consistent with an association between the two polymorphsms assessed and SVR. This association was particularly robust in carriers of the *+49 GG* genotype [45]. Our data do not replicate this findings since we found no significant associations between polymorphism in the *CTLA4* gene and SVR. Reasons for discrepancy may be seek in the lower number of patients in our study compared with that of other investigations, which suggest underpower. Genuine population differences may offer an additional explanation.

Polymorphism in the *ITPA* gene has been related with a benign erythrocyte enzymopathy, which is characterized by the accumulation of ITP in red cells. The affected patients may develop anemia when they are treated with purine analogues. Ribavirin is a purine analogue and previous studies have shown that *ITPA* genetic variants leading to ITPA deficiency are associated with ribavirin-induced anemia in HCV-treated patients [46]. This association has been replicated in studies performed in HCV-monoinfected [47]–[50] and in HCV-HIV co-infected [51] patients. In our Spanish cohort, however, we were unable to reproduce these findings, since no significant associations were observed between the two *ITPA* gene variants assessed and ribavirin-induced anemia and/or the need of ribavirin dose reduction. Additionally, we failed to find any significant association between *ITPA* SNPs and neutropenia. Also, we did not find associations between *ITPA* polymorphisms and thrombocytopenia, a finding that has been reported in two independent cohorts [52], [53]. As virological response is concerned, our data do not suggest any association between *ITPA* genetic variants and virological response. Our data therefore agrees with that reported by Chayama et al. [54], but differs markedly from that provided by Ochi [47] and Kurosaki [55]. It is unlikely that population differences could explain this discrepancy, since these three studies have been performed in Japanese.

We acknowledge that our work has some limitations that should be taken into account when interpreting the data. The number of patients assessed is low for a genetic association analysis and this may render our study underpowered for finding some significant associations. We believe, however, that studies performed with a phenotypically well-defined population such as ours may provide useful material for performing meta-analyses which could overcome issues of small sample size. Additionally, our cohort had a mixture of HCV genotypes. Since HCV genotype is a strong determinant of HCV treatment response, this could be a bias in our study. Despite these limitations, this is the first pharmacogenetic study arising from a randomised clinical trial performed in HCV-HIV co-infected patients and we believe that this design gives additional value to our findings.

In summary, in HCV-HIV co-infected patients treated with PegIFNα and ribavirin, SVR is associated with *IL28B rs8099917* polymorphism. Neutropenia and thrombocytopenia are associated with *SOCS3 r4969170* polymorphism.
